# Coakley on the Nose and Throat

**Published:** 1900-03

**Authors:** 


Coakley on the Nose and Throat.—The diagnosis and treatment of diseases of the nose, throat, naso-pharynx and trachea. For the use of students and practitioners. By Cornelius G. Coakley, M. D., Professor of Laryngology in the University and Bellevue Hispital Medical College, New York. In one handsome 12-mo. volume of 536 pages, with 92 engravings and two colored plates. Cloth, $2.75, net. Lea Brothers & Co., Philadelphia and New York.

This manual is intended to place before the student and practitioner a practical work on diseases of the nose and throat. Special attention has been given to examination, diagnosis and treatment. The pages devoted to hypertrophic rhinitis are unusually good, the various conditions met and the treatment best adapted for each being clearly and forcibly expressed. The arch operation for the correction of deviations of the septum is the one which the author believes will give the best results in the largest number of cases.

The differential diagnosis of tonsillitis and diphtheria, as given by the author, will be appreciated by the physician who can not always turn to the microscope. A special chapter has been devoted to therapeutics. I have tried a number of the prescriptions, and can testify to their value.

The book is clear, concise, well written and well printed.

